# Takotsubo syndrome with pulmonary embolism: a case report and literature review

**DOI:** 10.1186/s12872-018-0953-7

**Published:** 2018-12-10

**Authors:** Qi Jin, Qin Luo, Zhihui Zhao, Qing Zhao, Xue Yu, Lu Yan, Liu Gao, Zhihong Liu

**Affiliations:** Center for Pulmonary Vascular Diseases, Fuwai Hospital, National Center for Cardiovascular Diseases, Chinese Academy of Medical Sciences and Peking Union Medical College, 167 Beilishi Road, Xicheng District, Beijing, 100037 China

**Keywords:** Takotsubo syndrome, Pulmonary embolism, Literature review

## Abstract

**Background:**

Takotsubo syndrome (TTS) is an acute cardiac condition with reversible heart failure which is often triggered by psychological and physical stressful events. Although pulmonary embolism (PE) was reported as a trigger for TTS, the concurrence of TTS and PE has been rarely reported, let alone that triggered by PE. Here we describe a case of a postmenopausal female presenting with symptoms similar to myocardial ischemia, which may be caused by PE, and review the available literature that may help clinicians with their practice to similar situations since no published guidelines are available.

**Case presentation:**

An 86-year-old female was referred to the emergency department for unrelieved chest tightness, shortness of breath and back pain. Cardiac biomarkers were mildly elevated and electrocardiogram displayed pathologic Q-waves, ST-segment elevation and inverted T-waves. Unexpectedly, coronary angiography was in absence of obstructed coronary atherosclerosis or acute plaque rupture. Chest computed tomography illustrated multiple pulmonary emboli in bilateral pulmonary arteries. She had suffered from long-term right lower extremity pain and experienced a long railway journey with less activity. Both echocardiogram and cardiac magnetic resonance demonstrated regional hypokinesia of left ventricle. She received anticoagulant and diuretic therapies, three-month follow up after discharge revealed uneventful recovery without any pulmonary emboli or regional motion abnormalities, thus she was retrospectively diagnosed with TTS caused by PE.

**Conclusion:**

TTS and PE are scarcely concurrent and PE can exert as a potential trigger for TTS. TTS is easily misdiagnosed, actively seeking possible risk factors of TTS is in favor of early diagnosis and timely intervention. TTS with PE is reversible, timely and effective treatments ensure the best possible outcome.

**Electronic supplementary material:**

The online version of this article (10.1186/s12872-018-0953-7) contains supplementary material, which is available to authorized users.

## Background

Takotsubo syndrome (TTS) is characterized by transient left ventricular systolic dysfunction without evidence of obstructive coronary artery disease or acute plaque rupture. Postmenopausal women account for about 90% of cases, women > 55 years old have 4.8 times higher risks of developing TTS compared to those < 55 years old. It is frequently triggered by physically or emotionally stressful events and has similar clinical manifestations as acute coronary syndrome (ACS) [1, 2]. The pathogenesis of TTS is not well established, although pulmonary embolism (PE) has been listed as a potential contributor, TTS with PE is rarely described. Herein, we present a rare case of TTS triggered by PE which completely recovered within short time under anticoagulant and diuretic therapies. We also focus on the available case reports of TTS concurrent with PE since no treatment guidelines are available. This case report and literature review may promote better understanding of TTS and have great implications in clinical practice.

## Case presentation

An 86-year-old woman was referred to the emergency department (ED) for unrelieved chest tightness, shortness of breath and back pain for 6 h on November 7, 2016. Three hours before admission, she presented to her local hospital and was newly diagnosed with acute anterior myocardial infarction (MI), loading doses of aspirin and clopidogrel were orally taken but failed to relieve her symptoms. Five days prior, she had experienced chest tightness on exertion with shortness of breath and no back pain, and these symptoms resolved within a few minutes to two hours at rest and failed to draw her attention. Past medical history included previous pulmonary tuberculosis, besides, she had 40 years of smoking history but quit 16 years ago.

**Fig. 1:**
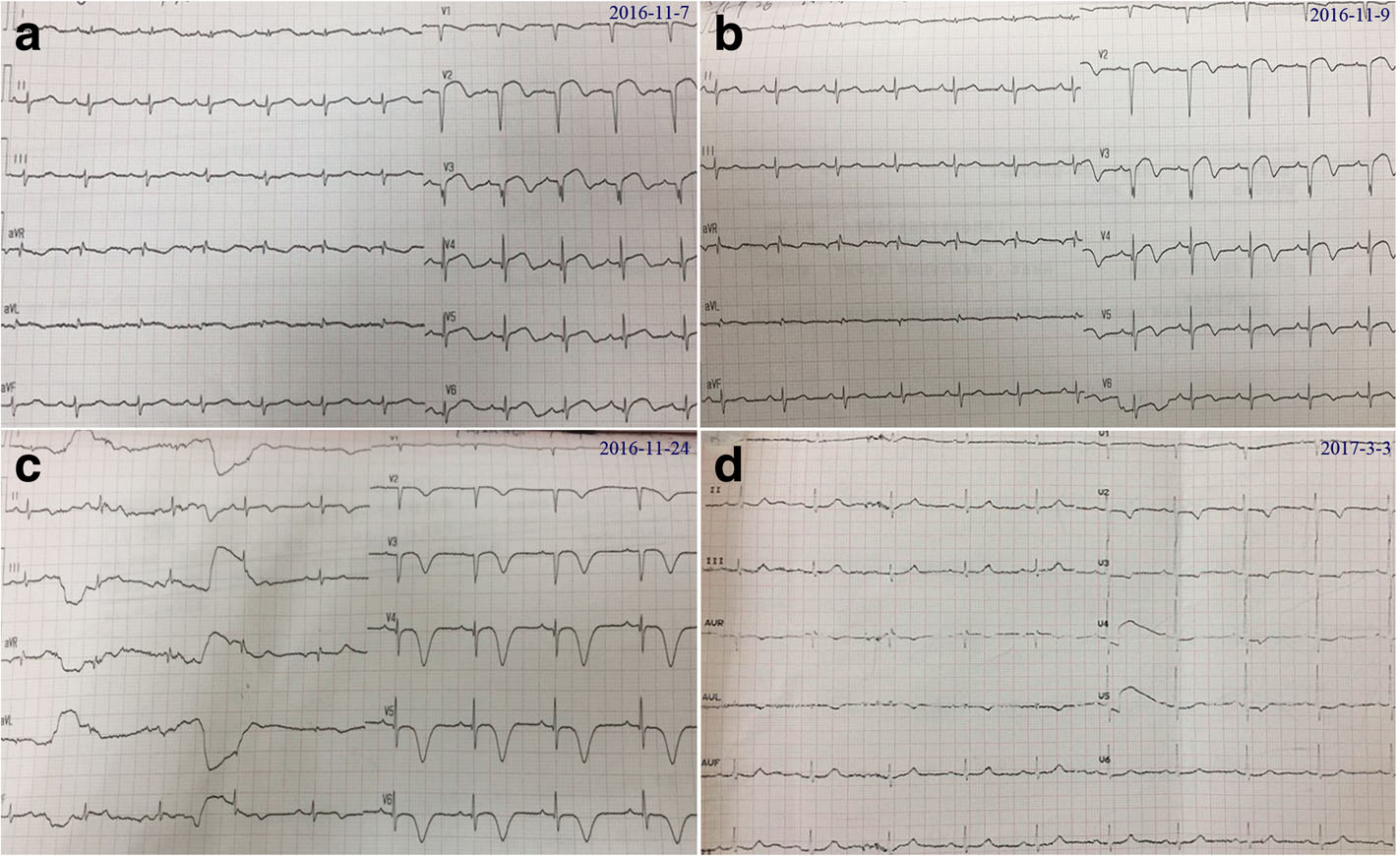
Dynamic ECG changes from admission to 3 months follow-up. **a** ECG on admission; **b** ECG on Day 2 after admission; **c** ECG at discharge; **d** ECG at 3 months follow-up

Physical examination revealed vital signs as follows: blood pressure 100/76 mmHg, heart rate 82 beats/min, respiratory rate 20/min. The cardiopulmonary examination was unremarkable, and no peripheral edema was present. The routine laboratory tests at ED revealed troponin I (cTnI) was 0.041 ng/ml (normal reference range 0–0.02 ng/ml), and creatine kinase-MB (CK-MB) was 6.54 ng/ml (normal reference range 0–4.99 ng/ml). No abnormalities were observed in complete blood count, renal and liver function tests. Electrocardiogram (ECG) showed abnormal Q-waves in leads I, aVL and V2-V9, ST-segment elevation in leads V2-V9, biphasic T-waves in V2-V9 and negative T-wave in V1 (Fig. 1a).

Echocardiogram was performed at ED admission, showing normal tricuspid annular plane systolic excursion and right ventricular diameter of 19 mm, an enlarged left ventricle (LV) of 53 mm at end-diastole with a reduced ejection fraction (EF) of 36% and decreased motion of the left ventricular anterior, anteroseptal, anterolateral wall and apex, and mild tricuspid regurgitation was observed with an estimated pulmonary artery systolic pressure (PASP) of 51 mmHg. Acute anterior wall and high lateral wall MI was initially diagnosed, thus emergency coronary angiography (CAG) was performed (Additional file 1: Video 1–2). Stable coronary plaques without signs of acute plaque rupture or coronary dissection were seen in the left anterior descending branch and right coronary artery. She was then sent to and managed in the coronary care unit after CAG. Dynamic changes of blood biomarkers and arterial blood gas results were summarized in *Table1*. Unexpectedly, CAG and dynamic alterations of cardiac biomarkers and ECGs seemed to rule out the initial diagnosis (Fig. 1*,* Additional file 1: Video 1–2*,* Table 1). Repeat echocardiogram on Day 1 displayed a normal-sized LV with left ventricular anterior, anteroseptal and apical hypokinesia and a left ventricular EF of 41%.

**Table 1:** Dynamic changes of biomarkers and blood gas results from admission to 3 months after discharge

Date	16.11.7	16.11.7	16.11.8	16.11.9	16.11.14	3 months
	(on admission)	(after CAG)				after discharge
CK-MB (ng/ml)	6.54 (< 4.99)	5.22 (< 4.99)	5.93 (< 4.99)	–	1.25 (< 4.99)	–
Myoglobin (ng/ml)	–	27.22 (< 70)	223.95 (< 70)	–	24.01 (< 70)	–
cTNI (ng/ml)	0.041 (< 0.02)	0.108 (< 0.15)	0. 067 (< 0.15)	0.047 (< 0.15)	0.033 (< 0.15)	0.002 (< 0.15)
D-Dimer (ug/ml)	–	12 (< 0.55)	10.28 (< 0.55)	2.59 (< 0.55)	1.77 (< 0.55)	0.32 (< 0.55)
NT-proBNP (pg/ml)	–	5727.8 (< 300)	4487.7 (< 300)	7286 (< 300)	3783.5 (< 300)	255.9 (< 300)
Blood Gases						
Oxygen Flow	–	3 L/min	–	3 L/min	3 L/min	–
pH	–	7.41	–	7.48	7.43	7.44
pCO2 (mmHg)	–	29	–	34	37	39
pO2 (mmHg)	–	86.3	–	89	87	75
SaO2 (%)	–	97.8	–	99	99	95

**Fig. 2:**
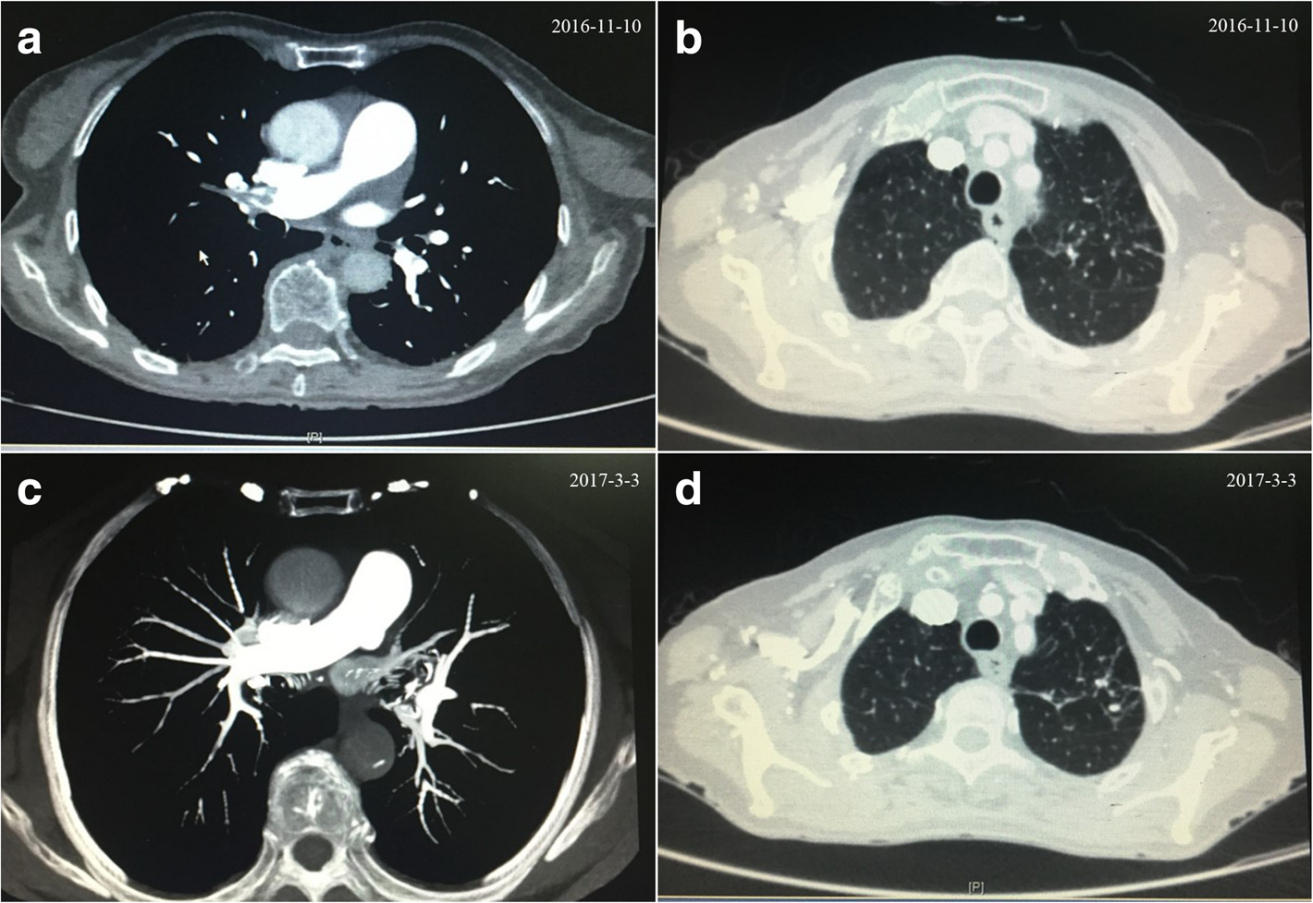
Comparison of chest CT on Day 3 after admission (**a** and **b**) and at 3 months follow-up (**c** and **d**)

By detailly inquiring medical history again, many years of right lower extremity pain, weakness of bilateral lower limbs for half a year and inactivity in the train for 10 h half a month ago were complained of. Since D-Dimer was extremely high, lower extremity venous ultrasound was done on Day 2, indicating multiple thrombi in the right intermuscular vein, dilation of bilateral deep femoral vein and spontaneous echo contrast. The following chest computed tomography (CT) illustrated multiple emboli in bilateral pulmonary arteries, calcified nodules in left upper pulmonary lobe, posterior segment of the right upper lobe and the anterior basement of the lower lobe (Fig. 2).

**Fig. 3:**
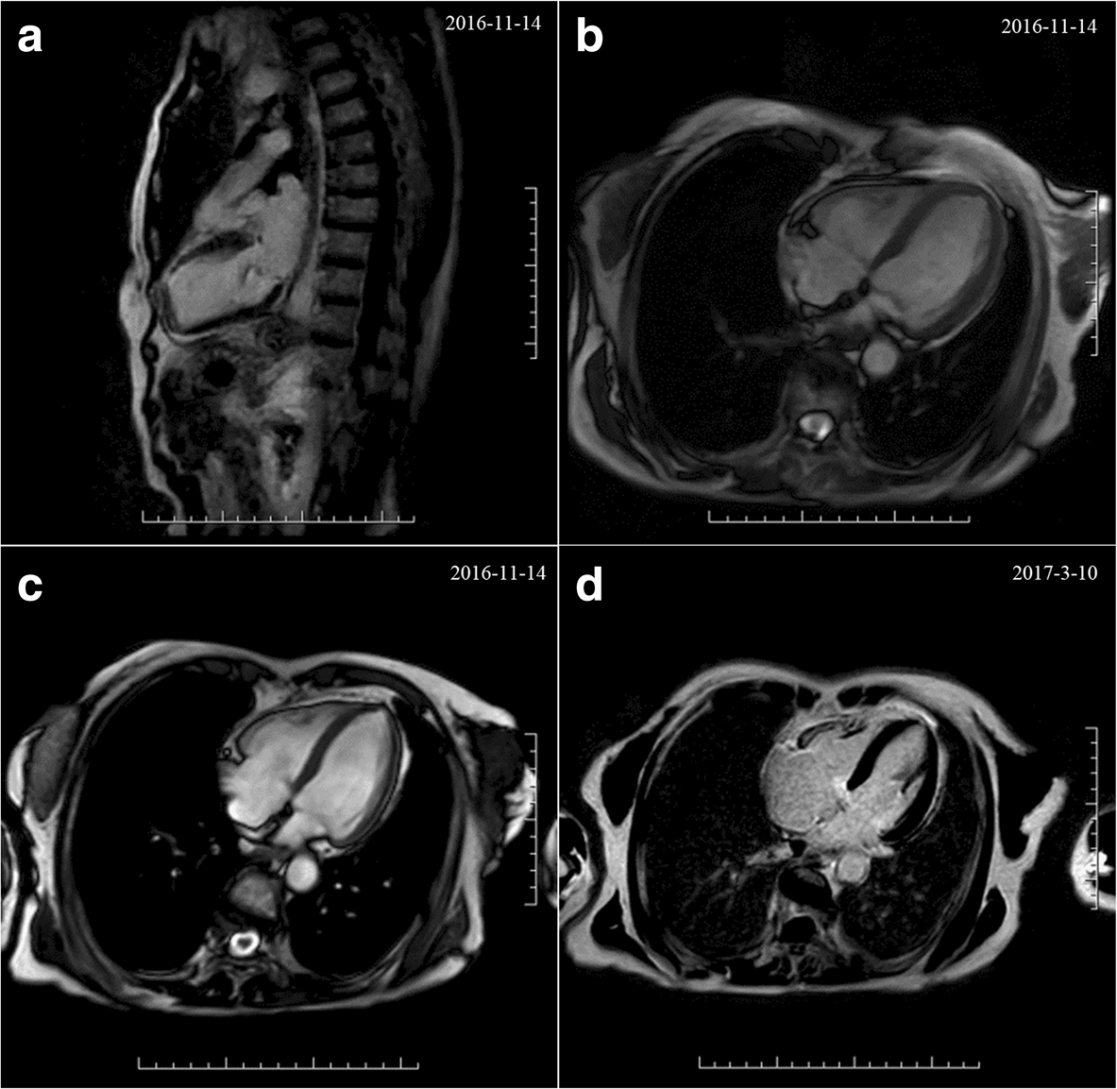
Comparison of CMR on Day 7 after admission (**a**-**c**) and at 3 months follow-up (**d**)

Cardiac magnetic resonance (CMR) (Fig. 3a-c*,* Additional file 1: Video 3–4) on Day 7 demonstrated paradoxical systolic motion of anterior left ventricular wall and akinesis of apex with an EF of 43%, and TTS could not be excluded. Filling defects in the right upper lobe and basal segments of the right lower lobe corresponded with pulmonary thromboembolism.

**Fig. 4:**
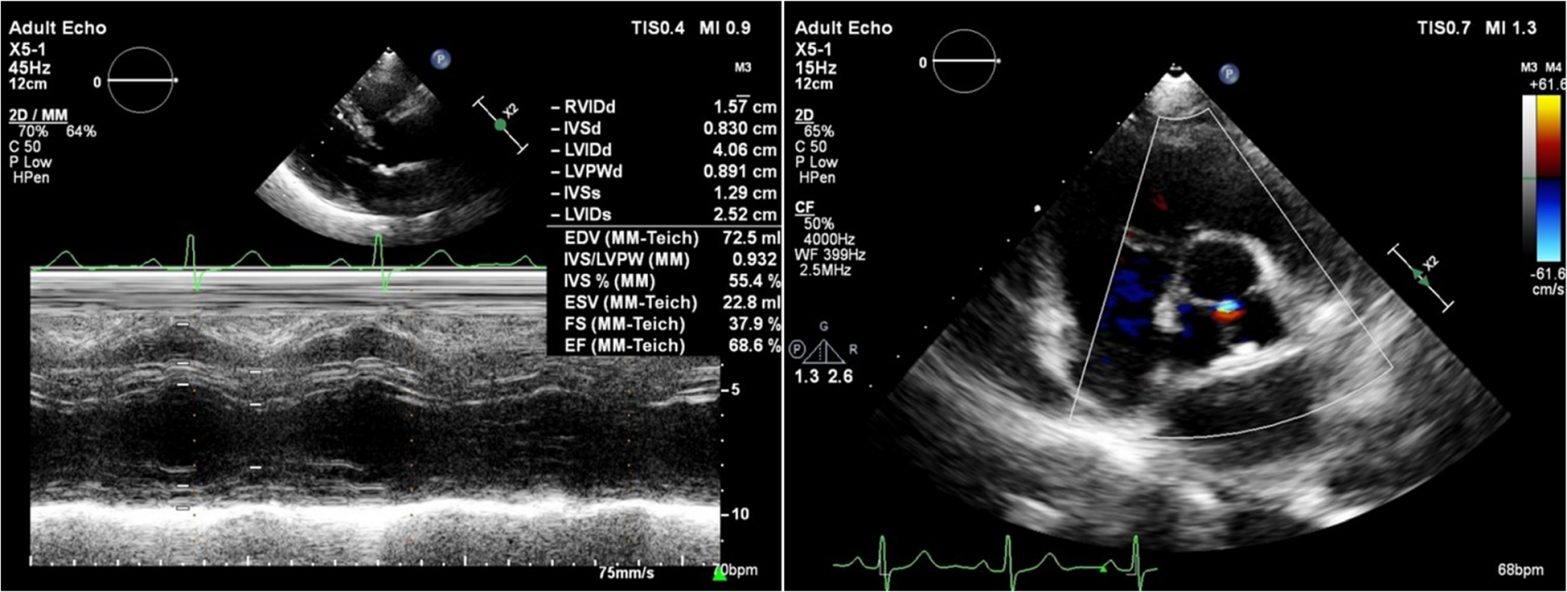
Echocardiogram at 3 months follow-up, showing normal systolic function

She received aspirin 100 mg qd, clopidogrel 75 mg qd, isosorbide dinitrate 10 mg tid, atenolol 3.125 mg bid, atorvastatin 20 mg qn, nicorandil 5 mg tid, furosemide 20 mg qd and spironolactone 20 mg qd and enoxaparin 0.3 ml qd on admission. Angiotensin converting enzyme inhibitors were not used due to low blood pressure. Dual antiplatelet therapy was terminated and enoxaparin was changed to 0.3 ml q12h while PE was suspected, and enoxaparin was switched to rivaroxaban 15 mg bid after one week, the latter was used for three weeks and was adjusted to 15 mg qd in consideration of her old age and bleeding risk. She was in a stable condition at 3 months follow-up after discharge, no chest tightness, chest pain or shortness of breath were complained of. Serological biomarkers and blood gas results were within normal ranges (Table 1)*.* Echocardiogram revealed normal-sized LV with an EF of 68%, mild aortic valve regurgitation and tricuspid regurgitation, but no regional wall motion abnormalities were detected (Fig. 4). No any evidence of PE was spotted in CT. CMR demonstrated improvement in left ventricular systolic function in comparison with the former (Fig. 3d*,* Additional file 1: Video 5–6). Hence, her final diagnosis was corrected as TTS with PE and bilateral deep venous thrombosis.

## Discussion and Conclusions

TTS is an acute reversible heart failure syndrome which was first described in 1990 and mainly occurs in postmenopausal women [3]. The pathological mechanism of TTS remains unclear, several possible theories have been postulated, such as catecholamine excess, coronary artery spasm, microvascular dysfunction and metabolic disturbance [2, 4].

Our patient is a postmenopausal woman, presenting with similar presentations to ACS. Abnormal ECG changes and elevated cardiac biomarkers on admission led to a suspected diagnosis of ST-elevation MI. However, urgent CAG along with dynamic ECG changes and biomarkers didn’t support the aforementioned diagnosis. Both repeated echocardiogram and CMR confirmed transient hypokinesia of left ventricular apex and regional left ventricular wall motion abnormalities. Besides, CMR didn’t support any signs of myocarditis or other cardiomyopathies. She made an uneventful recovery under treatments for three months without any elevated cardiac biomarkers, abnormal ECG changes and regional wall motion abnormalities. All in all, she was retrospectively diagnosed with TTS according to the Mayo Clinic diagnostic criteria, the most widely accepted criteria for clinical diagnosis of TTS [2].

TTS shares similar clinical presentations with ACS, thus it is quite easy to misdiagnose. Nevertheless, several significant differences exist between them. Near 90% of the patients with TTS were elderly postmenopausal women, but women had a lower incidence of ACS than men in all ages [5]. CAGs of TTS patients lack culprit atherosclerotic coronary artery lesions. Besides, TTS exhibits milder increase of CK-MB and cTnI compared to MI, cTnI levels of our patient were slightly elevated and presented a gradual declining trend. NT-proBNP level in TTS was significantly increased (mean > 4000 pg/ml), often 3–4times higher than that in ACS, reaching its peak value at 48 h after presentation [6, 7], its levels in our patient peaked at 7286 pg/ml 2 days after admission. It was demonstrated positive T waves in lead aVR and no negative T waves in lead V1 identified TTS with a sensitivity of 95% and a specificity of 97%, compared with ACS, TTS possessed greater numbers of leads with negative T-waves and greater maximal amplitude of negative T-waves [8]. Her ECG at discharge represented extensively deep inverted T-waves in V2-V6 and biphasic T-waves in leads I-II, most importantly, positive T-wave in lead aVR and no negative T-wave in lead V1 were observed.

PE is a fatal disease and is easily overlooked due to its nonspecific clinical presentation. Though D-Dimer and lower-extremity deep vein thrombosis (DVT) indicated high possibility of PE, no typical S1Q3T3 pattern was observed in ECGs of our patient, CT confirmed multiple pulmonary emboli in bilateral pulmonary arteries. Paradoxical embolism referring to an embolus that transits from right-to-left-sided cardiac chambers was considered, but no patent foramen ovale and thrombosis were seen in repeated echocardiography. Medical history indicated she might for a long period of time have suffered from DVT, a usual cause of PE. As D-Dimer levels dwindled, NT-proBNP levels reached a peak, positive T wave in lead aVR and no negative T wave in lead V1 appeared at her discharge. Thus, her PE may precede TTS, we regarded acute PE as a reversible cause of TTS. Its pathophysiological mechanism remains unclear, but extensive perfusion defect in lungs after PE was observed in lung perfusion scintigraphy, one pathophysiological hypothesis might be addressed to increased catecholamine levels during severe pain and elevated oxidative stress following perfusion defect within the lung.

PE was considered as a potential trigger for secondary TTS [4], but TTS with PE is rarely reported [9–14]. Herein, we retrospectively reviewed seven available case reports of patients with TTS and PE (Table 2). TTS with PE occurs frequently in older women who suffered from thrombotic diseases. All patients reported in literature were female, 6/7 patients were > 55 years old. Four of them had a history of DVT, other three experienced pyelonephritis, malleolar fracture surgery and inactivity when on a long-haul flight respectively, the latter two are common causes of DVT. CK-MB, cTnI and cTnT were mildly elevated, not as much high as those in patients with ACS, dynamic changes of these cardiac biomarkers may assist in differentiating TTS with PE from MI. ECG abnormalities were reported to be present in > 95% of TTS patients during the acute phase, they alone were not sufficient to distinguish TTS with PE from ST-segment elevation MI [3], ECGs in all reviewed cases displayed non-specific ST-T changes. Urgent CAG is a useful tool to rule out MI, no culprit coronary arteries were found in all reviewed patients. Echocardiogram or left ventriculogram should be considered first to verify suspected TTS since all reported cases showed transient hypokinesia or akinesia of regional wall motion. Anticoagulants, diuretics and β-blockers may be effective, and all patients got uneventful recovery with improvements in systolic function, indicating reversible manifestation and good prognosis of patients with coexistent TTS and PE.

**Table 2:** Case report review of takotsubo syndrome with pulmonary embolism

Year	Age (Years)	Sex	Risk Factor	Presentation	HR	BP (mmHg)	D-Dimer	CK	CK-MB	cTnI	cTnT	ECG	UCG	CAG	Left Ventriculogram	Thrombus Position	EF	Treatment	Recover Time and Follow-up	Reference
2011	79	F	DVT	pain in left lower extremity and shortness of breath	NA	NA	NA	NA	NA	NA	0.52 (0–0.03 ng/ml)	non-specific T-wave changes	akinetic apex, anterior and inferior septum	NA	NA	right pulmonary artery branches to the lingula and middle lobe (CT)	45%	heparin, ACEI, β- blockers	normal (follw-up after 6 months)	[9]
2011	68	F	DVT	right lower extremity pain and mild dyspnea during a gastroenterology exam	NA	NA	NA	NA	NA	0.95 (mildly elevated)	NA	poor R-wave progression with no evidence of ST elevation in the precordial leads.	global hypokinesis and apical ballooning	normal	NA	right middle lobe pulmonary artery (CTPA)	20%	diuretics, ACEI, β- blockers	several days	[10]
2012	65	F	pyelonephritis, no DVT	flank pain, vomiting and profuse perspiration	90	80/50	NA	1548 (50–228 μg/l)	23.1 (0–3.8 ng/ml)	NA	2.36 (0–0.1 ng/ml)	ST elevation in D1, aVL, V1-V3	apical and anterior hypokinesia	normal	akinesis of the ventricular apex	cephalic and posterior-basal segments of the left lung (lung perfusion scintigraphy)	35%	enoxaparin	several days	[11]
2013	38	F	fracture	chest discomforts, arrhythmia and shortness of breath after surgery	75	90/60	1572 (0–243 ng/mL)	NA	27 (0–3.6 ng/mL)	5.3 (0–0.1 ng/mL)	NA	ST depression in V3-V5	hypokinesia of mid/base segments of LV with hypercontraction of apical segments	no obstructive atherosclerotic diseases	NA	right lower lobe pulmonary artery, anterior and posterior basal segment arteries (CT)	47%	heparin, β-blockers and diuretics	normal (follw-up after 3 months)	[12]
2015	61	F	DVT	acute hypoxic respiratory failure	122	141/77	NA	2647 (elevated)	14.7 (elevated)	0.05 (elevated)	NA	ST elevation in anteroseptal leads	dilated RV and severe RV systolic dysfunction	normal coronary anatomy without obstruction	apical ballooning	emboli in the left and right main pulmonary arteries with extension into the upper and lower lobe branches (CT)	< 55%	heparin, clopidogrel, tPA, LMWH,warfarin	several days	[13]
2016	77	F	long-haul flight	dyspnea on exertion, orthopnea, and precordial chest tightness	83	92/58	4583 (FEU, normal < 750 μg/L)	NA	NA	NA	NA	anterolateral T-wave inversion	akinesia of LV apex	NA	NA	right segmental lower lobe artery (CTPA)	48%	LMWH, warfarin, bisoprolol, candesartan, aspirin, furosemide, spironolactone	normal (follow-up after 6 weeks)	[14]
–	86	F	DVT	chest tightness, shortness of breath and back pain	82	100/76	12 (normal < 0.55 ug/ml)	133 (0–200 IU/L)	6.54 (0–4.99 ng/ml)	0.041 (0–0.02 ng/ml)	NM	Q-waves in leads I, aVL and V2- V9, ST elevation in leads V2-V9, biphasic T-waves in V2-V9 and negative T-wave in V1	hypokinesia of the LV anterior, anteroseptal, anterolateral wall and apex	stable coronary plaques	NM	bilateral pulmonary arteries (CT)	36%	enoxaparin, rivaroxaban, aspirin, clopidogrel, β- blockers and diuretics	normal (follow-up after 3 months)	this case

*HR*: heart rate; *BP*: blood pressure; *CK*: creatine kinase; *cTnI*: troponin I; *cTnT*: troponin T; *ECG*: electrocardiogram; *UCG*: ultrasonic cardiogram; *CAG*: coronary angiography; *EF*: ejection fraction; *LMWH*: low molecular weight heparin; *F*: female; *DVT*: deep vein thrombosis; *NA*: not available; *ACEI*: angiotension converting enzyme inhibitors; *LV*: left ventricule; *RV*: right ventricle; *FEU*:fibrinogen equivalent unit; *NM*: not measured

TTS and PE are scarcely concurrent, here we present the first case of TTS caused by PE in China and for the first time review available similar literature. TTS with PE is easily misdiagnosed, clinicians should actively seek possible risk factors such as DVT for early diagnosis and timely intervention. TTS with PE is reversible, timely and effective treatments to eliminate the causes ensure the best possible outcome.

## Additional file

Additional file 1: Representative CAG and CMR. (PPTX 87696 kb)

## Abbreviations

ACS: Acute coronary syndrome
CAG: Coronary angiography
CK-MB: Creatine kinase-MB
CMR: Cardiac magnetic resonance
CT: Computed tomography
DVT: Deep vein thrombosis
ECG: Electrocardiogram
ED: Emergency department
EF: Ejection fraction
LV: Left ventricle
MI: Myocardial infarction
PASP: Pulmonary artery systolic pressure
PE: Pulmonary embolism
TnI: Troponin I
TTS: Takotsubo syndrome
