# Supplementary material for: Takotsubo syndrome with pulmonary embolism: a case report and literature review
Source: BMC Cardiovasc Disord. 2018 Dec 10;18:229. doi: 10.1186/s12872-018-0953-7 (PMC6288948; doi:10.1186/s12872-018-0953-7)
Supplement: Supplementary file 1 — Representative CAG and CMR. (PPTX 87696 kb) [file 12872_2018_953_MOESM1_ESM.pptx]

## Slide 1
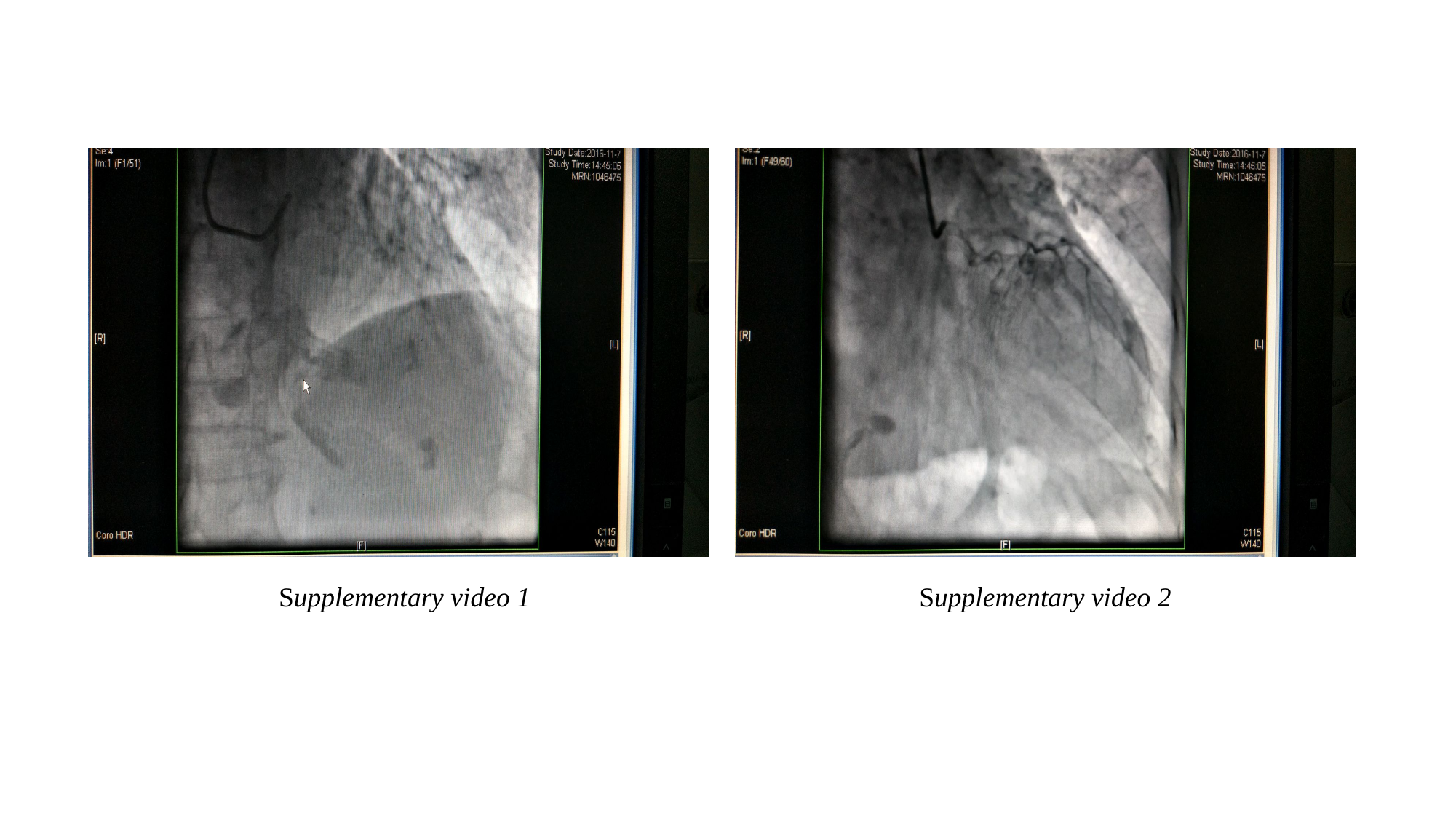

Supplementary video 1
Supplementary video 2

## Slide 2
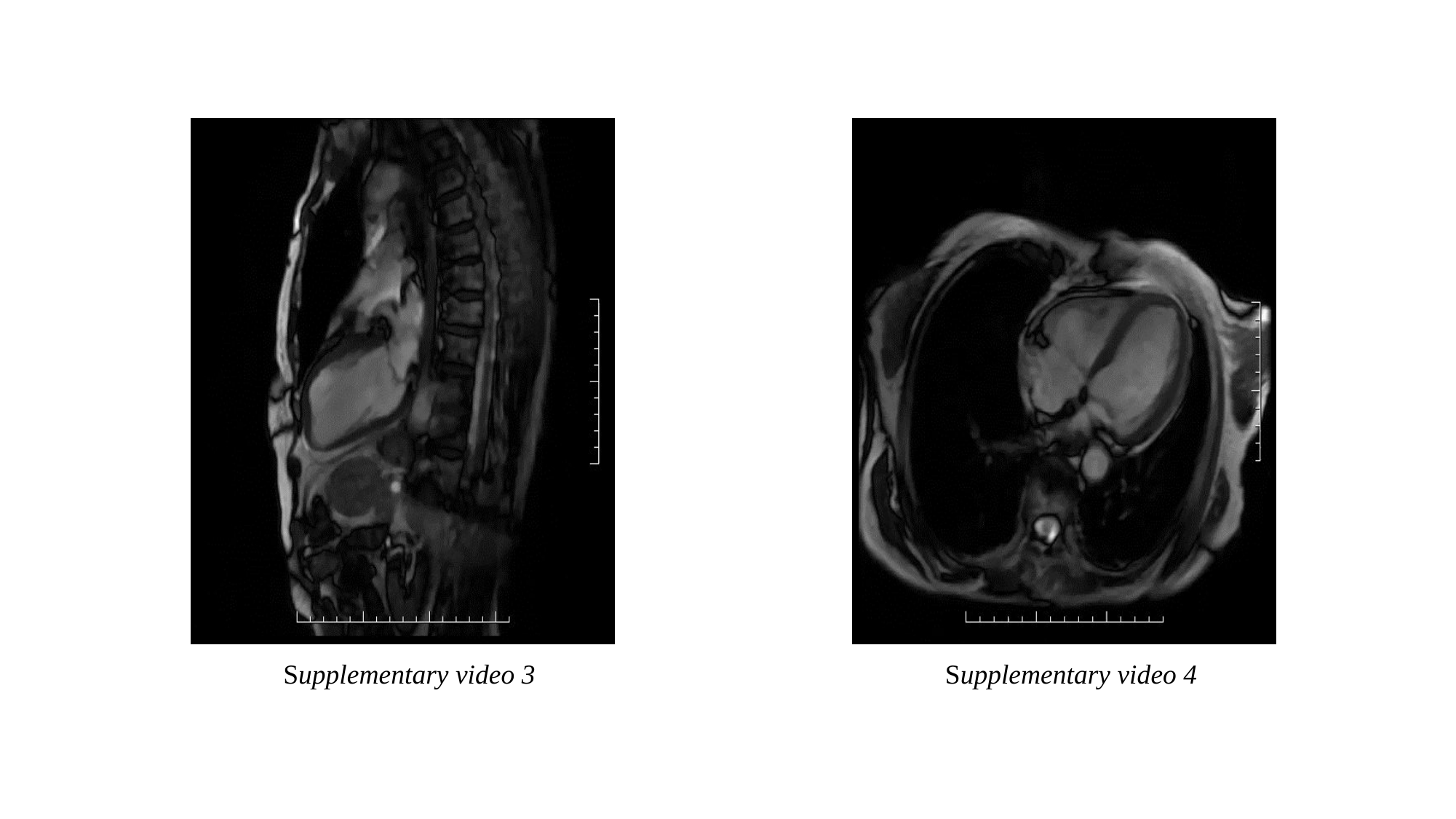

Supplementary video 3
Supplementary video 4

## Slide 3
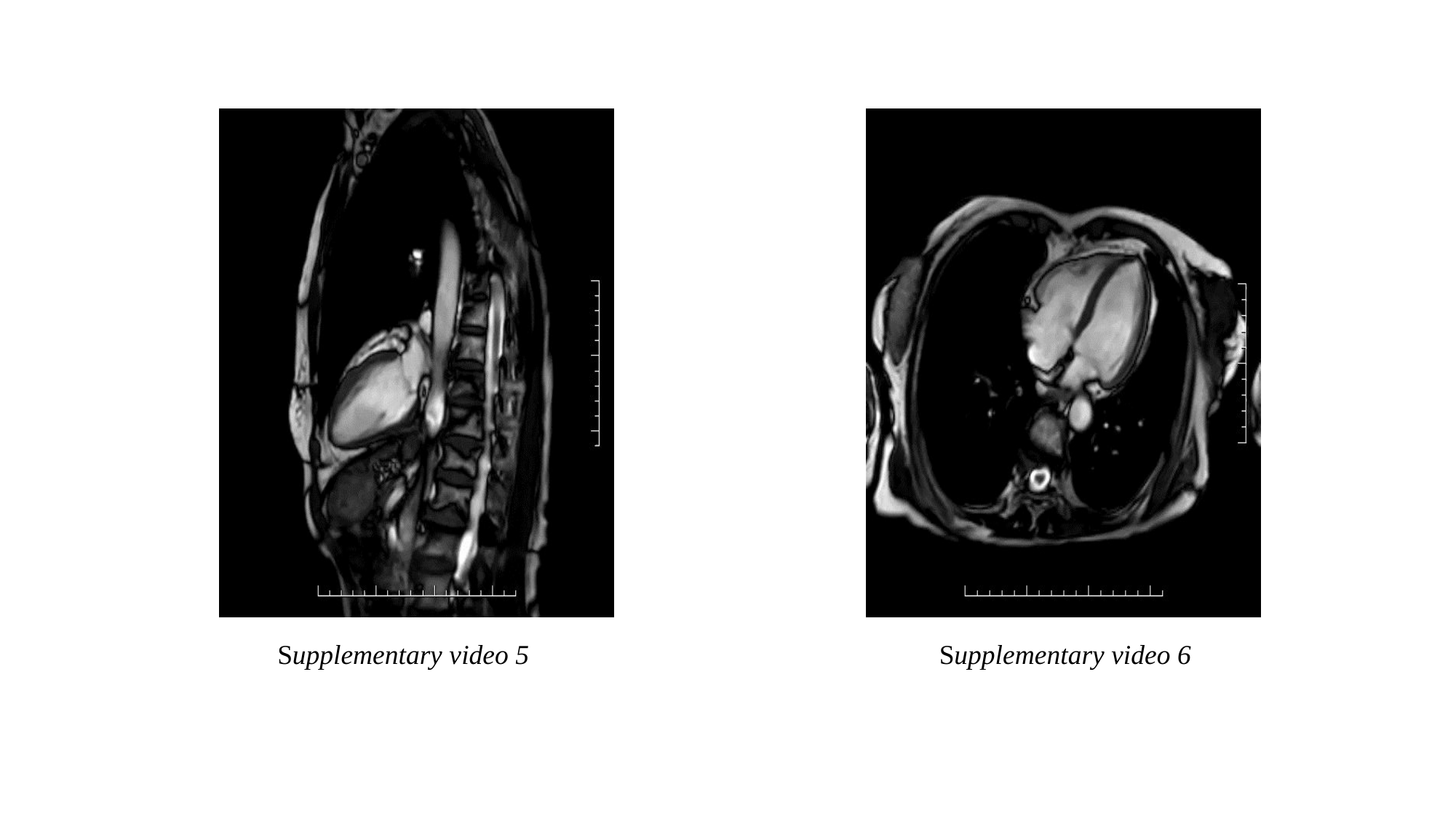

Supplementary video 5
Supplementary video 6
